# Can Artificial Intelligence (AI) be a Reviewer of a Medical Article?

**DOI:** 10.5146/tjpath.2024.13449

**Published:** 2024-05-18

**Authors:** Kutsal Yorukoglu

**Affiliations:** Department of Pathology, Dokuz Eylul University Faculty of Medicine, İzmir, Turkey

In this issue, a Letter to the Editor by Bolat B. et al. structured a mini study of prostate biopsies using the AI as the data extractor (1). I asked the following questions to the AI programmes that were used in that article :

1) Here’s a manuscript that I want you to review like a scientific journal reviewer with comments and recommendations.

2) Does it need a minor revision or major revision?

3) And I want you to make a decision on whether Turkish Journal of Pathology should accept this letter for publication or not.

**Meanwhile, the manuscript was peer-reviewed by human referees. Here are the Human and AI peer-review reports:**

**The Comments of the HUMAN referee:**

It is a relatively new and developing subject. There are many areas of AI use and this letter is important in terms of defining a new potential area.

Since it is not a study, it can be thought that it has shortcomings:

- Were the evaluated reports written in a standard format?

- Was this format synoptic?

- Was the terminology used the same?

- How many errors did the table created by artificial intelligence contain compared to the table created manually? What would be the impact of these errors on the study results? Would it lead to the wrong conclusion?

- Is the gold standard for evaluating the accuracy of the table created by artificial intelligence a manually created table?

- Instead of outsourcing this work to artificial intelligence, would a simple software that would enable the hospital information system to document this data provide easier and more accurate results?

- In Table 1, the authors state as “Parts of the extracted data are incomplete because the original report did not contain all the necessary information”. Is this true for all reports or some of them?

- Also, there exist stages as pT2N_M_ and (R1). What do those mean? Does N_ mean the specimen do not contain lymph node dissection? How was R1 stage generated? Does the AI generated R1 from “surgical margin positive” information, or do the authors added?

- Tumor volume is defined as <5% or 1%. Is this difference due to the difference of report format difference or AI construction?

- To see the human generated table would be helpful to evaluate the work done by AI.

Comment to Authors: As the authors state, artificial intelligence is open to development and will be a routine tool in daily practice in the near future. There is no limit to the areas of its use and it seems that just imagining will be enough. Although this article is not a study, it is important because it reveals a new view and points to new areas of use. It also provides guidance for investigating the question marks raised by this article.

Final Decision: This article needs major revision and should be reevaluated.

**The Referee reports of the ChatGPT and Google Bard:**

**Report of ChatGPT**

*After reviewing the manuscript titled “Large language models as a rapid and objective tool for pathology report data extraction,” submitted for publication in the Turkish Journal of Pathology, here are my comments and recommendations:*

*### General Assessment*

- **Originality and Relevance**: The manuscript addresses a significant and relevant issue in pathology research—efficient data extraction from pathology reports. The use of large language models (LLMs) such as ChatGPT and Google Bard for this purpose is innovative and aligns well with current trends in AI and medicine.

- **Clarity and Structure**: The paper is well-structured with a clear abstract, introduction, methods, results, and conclusion sections. The language is clear and professional.

*### Specific Comments*

*#### Strengths*

1. **Innovative Use of AI**: The application of LLMs to transform pathology reports into a structured format is a novel approach.

2. **Practicality**: The method proposed appears to be practical and could significantly reduce time and effort in data extraction processes.

3. **Comparative Analysis**: Comparing the performance of ChatGPT and Google Bard provides valuable insights into the practical application of these tools.

*#### Areas for Improvement*

1. **Sample Size and Diversity**: The study uses a limited sample size (10 reports) and focuses on radical prostatectomy specimens. Expanding the dataset and including different types of pathology reports could strengthen the results.

2. **Technical Details**: More technical details on the LLMs’ processing capabilities and limitations would be beneficial. For instance, how do these models handle complex medical terminologies or varying report formats?

3. **Error Analysis**: A more detailed analysis of errors or discrepancies in the AI-generated data, compared to manual extraction, would enhance the credibility of the findings.

4. **Discussion on Ethical and Privacy Concerns**: While de-identification of data is mentioned, a more thorough discussion on privacy, ethical considerations, and data security in the context of using AI for medical data extraction is necessary.

*### Recommendations for Revision*

- **Major Revision**: The manuscript requires significant enhancements, particularly in expanding the dataset, providing more technical and error analysis, and discussing ethical and privacy aspects.

*### Decision on Publication*

- **Decision**: Pending Major Revision. The manuscript is promising and aligns well with the journal’s scope, but requires major revisions before it can be considered for publication.

*### Additional Notes*

*- It would be beneficial to include case studies or real-world applications to showcase the practical utility of the proposed method.*

*- A section on future directions, discussing how this technology might evolve and its potential broader impacts on pathology and medical research, would be valuable.*

*The manuscript holds promise in advancing the use of AI in pathology and could contribute significantly to the field once the above-mentioned revisions are made.*

***Report of Google Bard***

*Sure, I have reviewed the manuscript you provided and here are my comments and recommendations:*

*Strengths:*

• *The authors propose a novel and potentially efficient method for pathology report data extraction using large language models (LLMs).*

• *The study is well-structured and includes a clear introduction, methods, results, and discussion sections.*

• *The authors acknowledge the limitations of their study, such as the small sample size and the need for further validation.*

*Weaknesses:*

• *The methods section is not very detailed, and it is unclear how the LLMs were trained and evaluated.*

• *The results are presented in a table format, but it would be helpful to include some additional quantitative data, such as the accuracy and precision of the LLMs.*

• *The authors do not discuss the potential ethical implications of using LLMs for pathology report data extraction, such as the risk of bias or privacy violations.*

*Overall:*

*This is a promising study that demonstrates the potential of LLMs for pathology report data extraction. However, the manuscript would benefit from some additional details and a more thorough discussion of the limitations and ethical implications.*

*Decision:*

*I believe that this manuscript could be publishable in the Turkish Journal of Pathology with some revisions. I recommend that the authors:*

• *Provide more details about the training and evaluation of the LLMs.*

• *Include some additional quantitative data in the results section.*

• *Discuss the potential ethical implications of using LLMs for pathology report data extraction.*

*Additional comments:*

• *The authors may want to consider using a different title that is more specific to their study. For example, “Using large language models for semi-automated data extraction from pathology reports: a pilot study”.*

• *The authors should carefully proofread the manuscript for any grammatical errors or typos.*

*I hope these comments and recommendations are helpful. Please let me know if you have any other questions.*

*Revisions needed:*

*This manuscript requires minor revisions before it can be accepted for publication.*

*Specific comments:*

• *The authors should provide more details about the training and evaluation of the LLMs. For example, they could specify what data was used for training, what metrics were used for evaluation, and how the LLMs were compared to other methods.*

• *The authors should include some additional quantitative data in the results section. For example, they could report the accuracy and precision of the LLMs for each data point, as well as the overall time savings achieved by using LLMs compared to manual data extraction.*

• *The authors should discuss the potential ethical implications of using LLMs for pathology report data extraction. For example, they could consider the risk of bias in the LLMs, the potential for privacy violations, and the impact on the job market for pathologists.*

*I hope these comments are helpful. Please let me know if you have any other questions*.

I think that the results obtained with the evaluation report created by artificial intelligence is not much different from peer-review human reports. But there are some differences that should be discussed. AI reports are longer and have indirect expressions. AI reports do not get to the core of the article and approaches from a general perspective. The ethical issue is one of them that was not included in the human referee report. However, the aim of this article is to obtain data for a study with ethical approval. There is no harm to the patient because the method used in data collection will be different for a study to be carried out with the approval of the patient or the ethics committee. The ethical dimension of the harm that may cause harm to patients by errors that may occur when artificial intelligence is used to collect data in the conduct of such a study is the subject of a very general discussion of artificial intelligence ethics and is far beyond the scope of this article, and even poses the danger of taking the targeted message in the wrong direction and out of the context in which it should be thought and discussed.

It is observed that both programs express the same comment in different ways. Does this mean that artificial intelligence is actually at the beginning of the road and has not yet gone beyond the given commands and revealed its own personality and interpretation? Does this make one think that artificial intelligence is far from its intended purpose?
